# Acute Pharmacological Effects of Oral and Intranasal Mephedrone: An Observational Study in Humans

**DOI:** 10.3390/ph14020100

**Published:** 2021-01-28

**Authors:** Esther Papaseit, Eulalia Olesti, Clara Pérez-Mañá, Marta Torrens, Francina Fonseca, Marc Grifell, Mireia Ventura, Rafael de la Torre, Magí Farré

**Affiliations:** 1 Clinical Pharmacology Unit, Hospital Universitari Germans Trias i Pujol (HUGTP-IGTP), 08916 Badalona, Spain; epapaseit.germanstrias@gencat.cat (E.P.); mfarre.germanstrias@gencat.cat (M.F.); 2 Department of Pharmacology, Therapeutics and Toxicology, Universitat Autònoma de Barcelona (UAB), 08193 Cerdanyola del Vallés, Spain; 3 Integrative Pharmacology and Systems Neuroscience Research Group, Neurosciences Research Program, Hospital del Mar Medical Research Institute (IMIM) and Universitat Pompeu Fabra (CEXS-UPF), 08003 Barcelona, Spain; eulaliaom@gmail.com (E.O.); rtorre@imim.es (R.d.l.T.); 4 Department of Psychiatry and Forensic Medicine, Institut de Neuropsiquiatria i Adiccions (INAD), Universitat Autònoma de Barcelona (UAB), 08003 Barcelona, Spain; mtorrens@parcdesalutmar.cat (M.T.); mffonseca@parcdesalutmar.cat (F.F.); marcgrifellguardia@gmail.com (M.G.); 5 Energy Control, Associació Benestar i Desenvolupament, 08041 Barcelona, Spain; mireia@energycontrol.org

**Keywords:** mephedrone (4-methylmethcathinone), novel psychoactive substances (NPS), psychostimulants, cathinones bath salts, oral administration, intranasal administration

## Abstract

Mephedrone (4-methylmethcathinone) is a synthetic cathinone with psychostimulant properties which remains one of the most popular new psychoactive substances (NPS). It is frequently used orally and/or intranasally. To date, no studies have evaluated the acute effects and pharmacokinetics after self-administration of mephedrone orally (ingestion) and intranasally (insufflation) in naturalistic conditions. An observational study was conducted to assess and compare the acute pharmacological effects, as well as the oral fluid (saliva) concentrations of mephedrone self-administered orally and intranasally. Ten healthy experienced drug users (4 females and 6 males) self-administered a single dose of mephedrone, orally (*n* = 5, 100–200 mg; mean 150 mg) or intranasally (*n* = 5, 50–100 mg, mean 70 mg). Vital signs (blood pressure, heart rate, and cutaneous temperature) were measured at baseline (0), 1, 2, and 4 h after self-administration. Each participant completed subjective effects questionnaires: A set of Visual Analogue Scales (VAS), the 49-item Addiction Research Centre Inventory (ARCI), and Evaluation of the Subjective Effects of Substances with Abuse Potential (VESSPA-SSE) at baseline, 1, 2, and 4 h after self-administration. Oral fluid and urine were collected during 4 h. Both routes of mephedrone self-administration enhanced ratings of euphoria and well-being effects and increased cardiovascular effects in humans. Although it was at times assessed that the oral route produced greater and larger effects than the intranasal one, concentrations of mephedrone in oral fluid and also the total amount of mephedrone and metabolites in urine showed that concentrations of mephedrone are considerably higher when self-administered intranasally in comparison to orally. Controlled clinical trials are needed to confirm our observational results.

## 1. Introduction

Mephedrone (4-methylmethcathinone) is considered to be the most popular synthetic cathinone drug, resembling the designer drug 3,4-methylenedioxymethamphetamine (MDMA, ecstasy) [1]. It is an amphetamine with an additional beta-ketone group [2,3]. Mephedrone acts as a releaser of monoamines similar to MDMA, but with greater relative potency to release dopamine versus serotonin compared with MDMA [4,5], indicating more stimulant-like properties. After emerging at the new psychoactive substances (NPS) drug market, mephedrone has remained present among certain recreational drug/NPS users and particularly among chemsex participants [6,7].

Mephedrone is most commonly available in powder form, but it is also available as tablets and capsules. Similarly to other psychostimulant drugs, mephedrone can be consumed via different routes. The predominant patterns of use are oral ingestion and nasal insufflation (snorting), although there are also reports of use by rectal insertion and intravenous/intramuscular injection. Because of the common desire to recapture the pleasurable initial high, the use of different routes and re-doses are frequent [8,9]. Users sometimes reported mixing oral and nasal routes, and re-dosing during single-use sessions in which the total doses per session typically reached 0.5–2 g, usually taken in every one or two hours [10]. In this respect, in regular mephedrone recreational users, mephedrone induces some undesirable sub-acute effects such as negative mood, fatigue, and physical symptoms [11]. Additionally, numerous fatal cases and non-fatal mephedrone intoxication cases attributable to high-dose use of mephedrone and to poly-drug use have been documented and attributed to potential interindividual differences in pharmacokinetics–pharmacodynamics [12,13,14,15,16]. Mephedrone and mephedrone metabolites have been detected in human plasma, urine, hair, and nails [17,18,19,20,21]. Until now, pre-clinical self-administration models using mephedrone intravenously and orally have evidenced that mephedrone produces psychomotor speed improvement and abuse liability, both typical psychostimulant properties [22,23,24]. Different metabolic disposition studies including human specimens suggest that mephedrone is metabolized in part by the CYP2D6 isoenzyme [25,26,27,28].

Despite the non-depreciable recreational use of mephedrone over the last years, there is limited scientific knowledge about its acute pharmacological effects and pharmacokinetics in humans [26,27,28,29,30,31,32,33,34] and anecdotal data related to the route of administration. Although as mentioned, mephedrone is frequently used via oral and/or intranasal routes and/or mixing them, no studies have evaluated the acute pharmacological effects of mephedrone in humans comparing both routes of administration. To date, the only three experimental studies conducted with humans have focused primarily on the physiological and subjective effects produced after oral mephedrone, a route of administration least often associated with abuse presumably due to its slow onset of effects [29,30,31]. After controlled administration, the onset of peak effects (E_max_) produced by oral mephedrone occur about 0.5–0.75 h after [29].

In comparison, recreational users reported that the maximum effects produced by intranasal mephedrone occur within 5 min [34], similarly to other drugs also used intranasally [35,36,37]. Recently, an experimental study in humans was performed after controlled intranasal administration of mephedrone (100 mg nasally insufflated) in healthy volunteers describing the profile of pharmacokinetics of mephedrone and its enantiomers, but no data about its acute effects were included in the results published [32,33].

To date, there have been no comparisons of mephedrone using different common routes of administration despite the recreational use of mephedrone. The main objective of the present study was to compare the acute effects after self-administration of oral (ingestion) and intranasal (insufflation) mephedrone in observational naturalistic conditions.

## 2. Results

Table 1 presents a summary of the physiological and subjective effects where at least one statistical difference in peak effect (E_max_) and/or AUC_0–4 h_ were found and includes time-course (T-C) points that showed significant differences.

Supplementary Figure S1 presented individual data of systolic blood pressure (SBP) in order to show the elevated variability of the acute effects.

Supplementary Table S1 shows significant T-C statistical differences of each route of administration in comparison to placebo. All subjects tolerated study procedures well.

There were neither significant adverse effects including hallucinations, psychotic episodes, nor any other psychiatric symptoms for oral or intranasal mephedrone self-administration during the experimental session.

### 2.1. Physiological Effects

Regarding physiological effects, both oral and intranasal mephedrone self-administration produced an increase in SBP, DBP, HR, and T (see Table 1, Supplementary Table S1 and Supplementary Figure S1). Comparisons of the two routes of administration revealed no significant differences for E_max_, AUC_0–4 h_, and T-C in vital signs except for cutaneous T (E_max_). T-C comparison to baseline revealed significant differences at 1 and 2 h after oral self-administration for SBP, DBP, and HR, whilst after intranasal self-administration differences were found only at 1 h in comparison to baseline for DBP, HR, and T.

### 2.2. Subjective Effects

**Figure 1:**
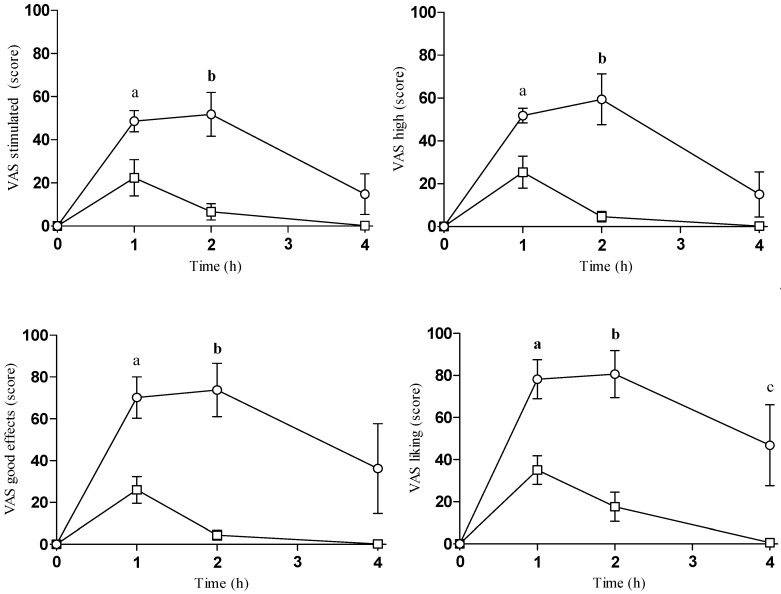

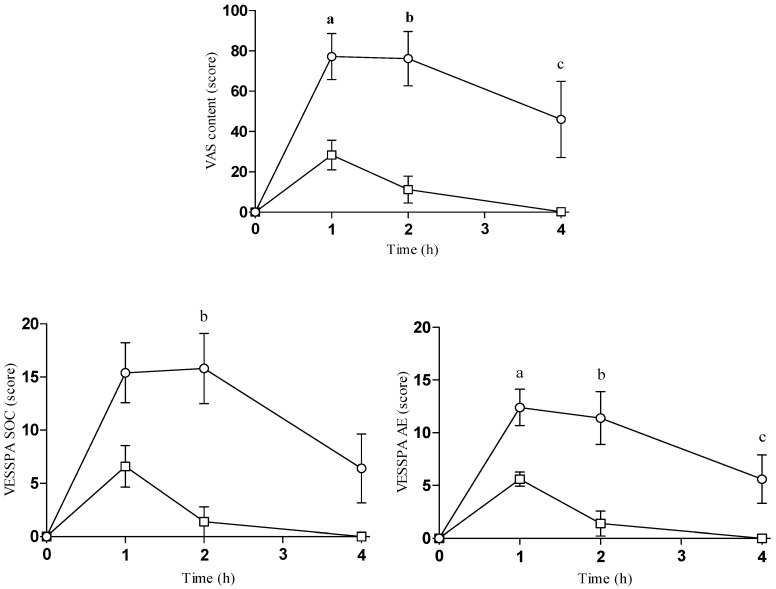
Summary of the course of physiological and subjective effects of mephedrone after oral and intranasal self-administration (○ oral mephedrone (*n* = 5); □ intranasal mephedrone (*n* = 5)). Statistical differences between oral and intranasal are presented as “a” *p* < 0.05, “**a**” *p* < 0.01 (time 1 h), “b” *p* < 0.05, “**b**” *p* < 0.01 (time 2 h), “c” *p* < 0.05, “**c**” *p* < 0.01 (time 4 h). See text for abbreviations.

Both oral and intranasal mephedrone increased subjective drug effects (VAS, ARCI, and VESSPA-SEE) (see Table 1, Supplementary Table S1 and Figure 1). The comparison of the two routes of administration showed significant differences for stimulant-like and pleasurable effects for both E_max_ and AUC_0–4 h_. T-C comparison between oral and intranasal mephedrone showed significant statistical differences for stimulated, high, good effects, liking, and content feelings at 1, 2, and/or 4 h after administration.

After oral mephedrone, T-C comparison to baseline showed significant differences for intensity, stimulates, high and good effects at 1 and 2 h, whilst for liking and content, differences were detected in all times evaluated. In contrast, after intranasal self-administration T-C comparison to baseline only showed significant differences for intensity, stimulates, high, good effects, and content at 1 h and for liking at 1 and 2 h, respectively.

Both oral and intranasal mephedrone produced mild changes in perceptions, but not hallucinations, although no statistically significant differences were detected among routes of administration except for different body feeling (AUC_0–4 h_).

With respect to the ARCI questionnaire, mephedrone self-administered orally and intranasally produced an increase in all the subscales evaluated. The most marked increases were observed in scores for the MBG (euphoria), BG (intellectual efficiency and energy), and A (amphetamine) subscales. When comparing both routes of administration, statistical differences were detected only for the MBG subscale in E_max_ and AUC_0–4 h_.

In comparison to baseline, statistical differences were shown in several T-C points after oral self-administration at 1 h and 2 h for MBG and BG subscales, and at 1, 2, and 4 h for A subscales, and also after intranasal self-administration at 1 and 2 h for PCAG, BG, and A subscales, and at 1h for the MBG subscale.

Regarding the VESSPA-SEE questionnaire, mephedrone increased all the subscales regardless of the route of administration except for the CP (changes in perception) subscale for intranasal mephedrone. Comparing both routes, statistical differences were observed in E_max_ and AUC_0–4 h_ for the SOC (pleasure and sociability) and ACT (activity and energy) subscales and only in AUC_0–4 h_ for PS (psychotic symptoms) subscale. Whilst for T-C, statistical differences were found only in ACT scores in all points.

T-C comparison at baseline revealed significant differences at 1 h for the PS subscale, at 1 and 2 h for ANX and SOC subscales, and at 1, 2, and 4 h for ATC subscales after oral self-administration, and at 1h for SOC and ACT subscales, and at 1 and 2 h for ANX subscales after intranasal self-administration.

### 2.3. Oral Fluid Concentrations of Mephedrone

**Figure 2:**
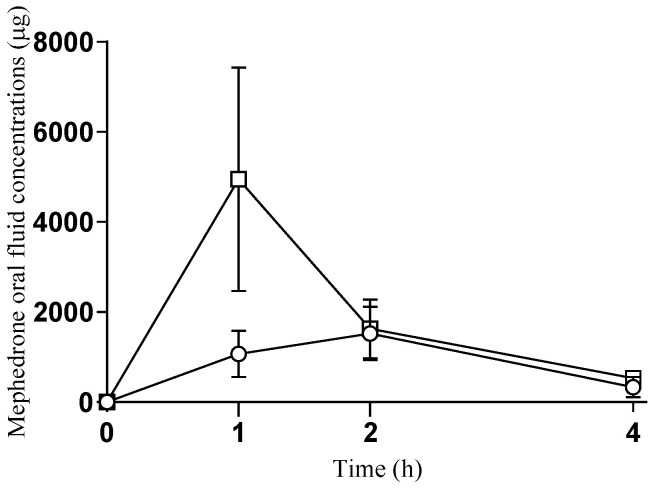
Time-course of mephedrone oral fluid concentrations after oral and intranasal self-administration (○ oral mephedrone (*n* = 5); □ intranasal mephedrone (*n* = 5)).

The oral fluid (saliva) T-C concentrations curve for mephedrone is shown in Figure 2 and Supplementary Figure S2 (individual data).

After self-administration of oral mephedrone, concentrations of mephedrone in oral fluid increased rapidly, reaching a peak 2 h after ingestion, and decreased at 4 h. Mean maximum concentration (C_max_) values of 1571 ± 1367 ng/mL (range 18–2999 ng/mL) were obtained at a T_max_ of 2 h following drug administration. The AUC_0–4 h_ was 3686 ± 3443 ng·h/mL (range 61–7593 ng·h/mL). At 4 h, all subjects presented mephedrone concentrations except for one subject that had no detectable concentrations.

After self-administration of intranasal mephedrone, oral fluid concentrations of mephedrone increased rapidly, reaching a peak 1 h after ingestion, and then rapidly decreased at 4 h. C_max_ values of 4950 ± 5545 ng/mL (range 1091–14,525 ng/mL) were obtained at a T_max_ of 1 h following drug administration. At 4 h, mephedrone concentration was 9 times lower (4–41 times) in comparison to C_max_. The AUC_0–4 h_ was 7917 ± 7717 ng·h/mL (range 1633–20,918 ng·h/mL). Oral fluid mephedrone concentrations varied considerably among oral and intranasal doses and subjects. No significant differences between oral and intranasal mephedrone were found for C_max_, AUC_0–4 h_, and T_max_ (Table 2).

### 2.4. Urinary Concentrations of Mephedrone and Metabolites

**Figure 3:**
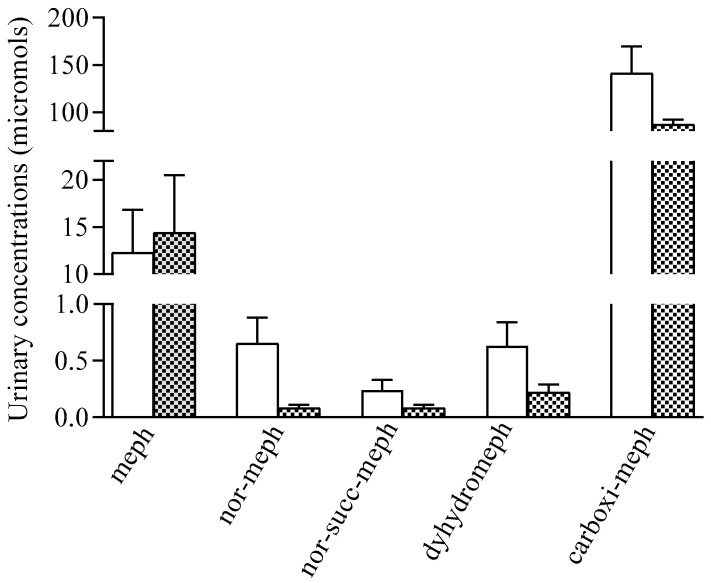
Urinary concentrations of mephedrone after oral and intranasal self-administration (unfilled bar: Oral mephedrone (*n* = 5); filled bar: Intranasal mephedrone (*n* = 5)).

Recovery of mephedrone and its metabolites nor-mephedrone, dihydro-mephedrone, 4-carboxy-mephedrone, and succinyl-nor-mephedrone in urine in the 0–4 h period post self-administration is shown in Figure 3. The profile of metabolites recovered in urine was similar for all doses tested, and for the oral doses it was congruent with previous data published [15].

## 3. Discussion

The overall purpose of this study was to describe the acute effects of oral and intranasal mephedrone in naturalistic conditions and to compare the two most important routes of its administration.

The present findings show that mephedrone self-administered in observational naturalistic conditions induced acute effects that are similar to those produced under experimental conditions [29,30]. Consistent with these results, mephedrone produced similar effects on the majority of physiological and subjective measures. Furthermore, both routes of mephedrone administration (oral and intranasal self-administration) enhanced ratings of euphoria and increased cardiovascular effects.

With respect to the pharmacological effects after oral self-administration of mephedrone, the magnitude and maximum intensity of the pharmacological effects are in accordance with those observed under controlled conditions. Overall, peak effects were observed between 1–2 h and returned to baseline 3–4 h after drug administration [29,30]. In relation to intranasal mephedrone, as mentioned initially, there is no previously published pharmacodynamic data to compare with. In general terms, the intranasal self-administration of mephedrone produces acute pharmacological effects similar to those produced by oral mephedrone. The most remarkable result of this study showed that, at times assessed (1 and/or 2 h), mephedrone oral self-administration in comparison to intranasal self-administration produced greater and larger effects on some subjective measures (e.g., ratings of VAS and several subscales of ARCI and VESSPA). Nonetheless, as would be expected, mephedrone, similarly to other psychostimulant drugs that are also usually used by the intranasal route (insufflation), dilated the vascular-rich areas of the intranasal cavity and pulmonary network, thus increasing the absorptive surface area and allowing for more rapid entry of the drug into the bloodstream, producing fast and reinforcing effects [37]. This well-known factor could justify the fact that the punctuation of subjects who self-administered intranasal mephedrone was lower than those who self-administered orally, because the first evaluation (at 1 h) was conducted once the maximum subjective effects were induced (several minutes after self-administration, which was not assessed).

According to previous published results of mephedrone pharmacokinetics in plasma after controlled intranasal administration, mephedrone showed rapid absorption with a mean T_max_ of 0.88 ± 0.35 h [32]. Besides, this T_max_ in plasma was slightly shorter in comparison to the plasma T_max_ of 1.2 h after controlled oral administration of 200 mg of mephedrone by our research group [29]. Again, these data point to faster acute pharmacological effects of intranasal mephedrone compared to oral mephedrone.

Additionally, both results obtained from concentrations of mephedrone in oral fluid and from the total amount of mephedrone and metabolites in urine confirm that concentrations of mephedrone are considerably higher after intranasal self-administration in comparison to oral self-administration. As expected, mean oral fluid concentrations of mephedrone at 1 h post administration was 4.6 times higher after intranasal than oral administration (4950 ng/mL versus 1070 ng/mL, respectively), achieving by both routes similar concentrations at 2 h.

In urine, again mephedrone concentrations were higher after intranasal than oral administration of mephedrone. In relation to mephedrone metabolites after oral self-administration, all metabolites were detected with a similar profile of recovery in comparison with a previous study [17], whilst there are no previous data for intranasal mephedrone.

The relevance of our results for intoxication cases is limited because usually concentrations in different biological samples have documented great variability. To date, urine concentrations have been analyzed in several intoxications and fatality cases of mephedrone, whilst there is no data about oral fluid ones. Urinary concentrations reported in clinical trials or mild intoxications are in the range of concentrations measured in our study [11,17,38].

Finally, there were a number of limitations presented by this study design. The main limitations associated with the study are the naturalistic-observational design, that doses varied across subjects and were different in subjects using intranasal vs. oral route and the number of time-point measures and their time interval. This last limitation is particularly important for the evaluation of fast acute effects. It did not permit us to accurately know the real maximal or peak effect/concentration times particularly for intranasal mephedrone, which will need more frequent and earlier evaluations. Other limitations to consider are the non-placebo-controlled design, because participants selected the dose and the route of administration according to their preferences and previous experiences (expectancy bias), a limited number of subjects (lack of statistical power in some measures). Furthermore, the effects reported by participants could have influenced the recreational setting. Finally, we did not collect data on genetic polymorphism of CYP2D6 that can influence the pharmacokinetics and effects of the substance.

However, the strengths should be remarked on: The participation of female subjects, the dose selection by the participants according to their preferences, the inclusion of two different routes of administration, effects previously experienced with the same or similar psychoactive substances, the recreational scenario, and the use of validated methodology using in controlled studies (rating scales, questionnaires) and analytic techniques.

Preliminary data from this observational study have pointed out for the first time that mephedrone profiles in real conditions may vary considerably depending on the route of administration due to the dose administered and the interindividual differences in pharmacodynamic-pharmacokinetics. Thus, it is not possible to make valid conclusions and comparisons, and controlled clinical trials are needed to confirm our observational results.

## 4. Materials and Methods

### 4.1. Participants

Ten healthy subjects were selected (4 females and 6 males). Participants were recreative drug users who had experience with amphetamines, ecstasy, mephedrone, and/or cathinones at least once in their lifetimes without experiencing previous serious adverse reactions.

Exclusion criteria included a history of any serious medical or psychopathological disorder including substance use disorder (except for nicotine), a previous serious adverse reaction with users of amphetamines, ecstasy (MDMA), mephedrone, and cathinones, and use of chronic medication. Participants were recruited by word-of-mouth and snowball sampling through the harm reduction, non-governmental organization Energy Control (ABD). The protocol was approved by the Clinical Research Ethics Committee. The study was conducted in accordance with the Declaration of Helsinki recommendations. All the participants were fully informed, both orally and in writing, about the study characteristics. All of them indicated their agreement to participate and signed an informed consent prior inclusion. Subjects were financially compensated for their participation.

### 4.2. Design and Treatments

The study was conducted according to a non-controlled prospective observational study with minimal intervention in subjects who self-administered mephedrone orally or intranasally. Similarly to previous naturalistic observational studies evaluating acute effects of other NPS, the methodology including evaluations and procedures were similar [39,40]. Each subject participated in one session. Treatment consisted of oral or intranasal self-administration of mephedrone that they brought to the testing site themselves, which they had obtained from an unknown source. Although no information was available about the synthesis of the drug, similar capsules tested by Energy Control, a harm reduction organization that provides a Drug Checking Service for users, showed that the substance contained mephedrone at 95% purity with no toxic adulterants. A gas chromatography associated with mass spectrometry (GC/MS) was previously used by the mephedrone analysis. The method used permits to check for most common drugs of abuse including most of the NPSs and to know the exact purity of mephedrone in the powder to prepare dosing by a precision scale [29]. The dose of oral and intranasal mephedrone self-administered was selected by the participants based presumably on their previous experience. Five subjects self-administered one dose of mephedrone orally, the mean mephedrone dose was 150 mg (100–200 mg) (1 female ingested 100 mg, 2 females and 1 male ingested 150 mg, 1 male ingested 200 mg), and five subjects self-administered one dose of mephedrone intranasally, the mean mephedrone dose was 70 mg (50–100 mg) (3 males insufflated 50 mg and 2 males 100 mg). All the selected doses were well tolerated, and no serious adverse events were observed. No local tissue damage to the nostrils or any other potential acute medical complication after snorting was reported.

### 4.3. Procedures

Prior to the study session, the participants underwent a general medical examination and a psychiatric interview. They received training with respect to questionnaires used in the study. Upon arrival, they were questioned about any event that could affect their participation and any drug use 2 days prior to the session. Participants were not allowed to consume alcohol or beverages containing caffeine the previous 24 h. The session took place on the same day at a private club with ambient music and participants could talk, read, or play table games during the session and interact in exception to the evaluation times. Moreover, they were instructed not to talk about the effects of the substance during the session. Assessments were performed at baseline (pre-dose) and 1, 2, and 4 h after oral or intranasal self-administration of mephedrone. The experiment was conducted from 15:00 to 21:00 h. Earlier assessment (<1 h) could not be carried out due to the setting of consumption. Urine spot samples were collected at baseline (pre-dose) to exclude prior substance drug use (benzodiazepines, barbiturates, morphine, cocaine, amphetamines, methamphetamine, MDMA, marijuana, phencyclidine) with Instant-View, Multipanel 10 Test Drug Screen (Alfa Scientific Designs Inc., Poway, CA, USA). Self-administration of mephedrone took place around 16.00 h. At each time point of the session, the sequence of procedures was: Physiological measures, oral fluid collection, subjective effects questionnaires, and urine recollection. During entire study session, a psychiatrist was present and adverse effects were assessed.

### 4.4. Physiological Effects

Physiological effects including non-invasive systolic blood pressure (SPB), diastolic blood pressure (DBP), and heart rate (HR) were determined with an Omron monitor at baseline and 1, 2, and 4 h after administration. Cutaneous temperature was measured simultaneously.

### 4.5. Subjective Effects

Subjective effects of mephedrone were measured at baseline and at 1, 2, and 4 h after self-administration using different scales and questionnaires. A set of Visual Analog Scales (VAS) (100 mm, from “not at all” to “extremely”) were used to measure rate intensity; stimulated; high; good effects; liking; content; changes in colors; changes in shapes; changes in lights; hallucinations—seeing of lights or spots; hallucinations—seeing animals, things, insects, or people; changes in hearing; hallucinations—hearing sounds or voices; different body feeling; unreal body feeling; changes in distances; different surroundings; unreal surroundings; confusion; fear; depression or sadness; drowsiness; dizziness; bad effects; headache; nausea; vertigo; breathing difficulty; and face flushing [29,39,40,41,42]. The 49-item Addiction Research Centre Inventory (ARCI) short form, a validated instrument that includes subscales related to drug sedation (pentobarbital chlorpromazine-alcohol group, PCAG), euphoria (morphine-benzedrine group, MBG), dysphoria and somatic symptoms (lysergic acid diethylamide group, LSD), intellectual efficiency and energy (benzedrine group, BG), and d-amphetamine like effects (A) that evaluate subjective effects produced by psychoactive drugs [38,39]. The Evaluation of the Subjective Effects of Substances with Abuse Potential (VESSPA-SE) questionnaire that includes subscales related to sedation (S), psychosomatic anxiety (ANX), changes in perception (CP), pleasure and sociability (SOC), activity and energy (ACT), and psychotic symptoms (PS) that measures changes in subjective effects caused by different drugs including stimulants and psychedelics [29,43].

### 4.6. Urinary Concentrations of Mephedrone and Metabolites

Urine samples were collected at baseline (0 h) and during the entire session (0–4 h). Urine was stored at −20 °C until analysis. Urinary samples were analyzed following a previously reported validated method based on liquid chromatography tandem–mass spectrometry (LC-MS/MS). Mephedrone and its main metabolites, nor-mephedrone, dihydro-mephedrone, nor-succinyl-mephedrone and carboxy-mephedrone were quantified [44,45].

### 4.7. Oral Fluid Concentrations of Mephedrone

Oral fluid samples were collected with Salivette^®^ tubes at 0 h (baseline), 2, and 4 h after mephedrone self-administration. After collection, samples were centrifuged and frozen at −20 °C until analysis. Mephedrone concentrations were analyzed by a validated LC-MS/MS [39,40]. A Mephedrone chromatogram (one participant that self-administrated orally 150 mg of mephedrone and one participant that self-administrated 50 mg intranasal) and chromatograms of the internal standard used for the previous samples (Mephedrone-d3) were available in Supplementary Figure S2 and linearity parameters of the oral fluid methodology in Supplementary Figure S3.

The oral fluid (saliva) T-C concentrations curve for mephedrone is shown in Figure 2 and Supplementary Figure S4 (individual data).

### 4.8. Statistical Analysis

Differences with respect to baseline were calculated for physiological (SBP, DBP, HR, and T) and subjective effects (VAS, ARCI, and VESSPA). Maximum effects (E_max_) were determined and the area under the curve of the effects (AUC_0–4 h_) was calculated using the trapezoidal rule by the Pharmacokinetic Functions for Microsoft Excel (Joel Usansky, Atul Desai, and Diane Tang-Liu, Department of Pharmacokinetics and Drug Metabolism, Allergan, Irvine, CA, USA).

To study possible differences between doses, a one-way analysis of variance (ANOVA) test including all doses for each route of administration as a factor was used for E_max_ and AUC_0–4 h_.The results showed <15% of statistically significant differences among doses for each route of administration. Therefore, it was decided to consider all doses for each route of administration globally, and Student’s *t*-Test for paired sample was conducted for E_max_ and AUC_0–4 h._

To compare the time course (T-C) of effects of mephedrone between the two routes of administration, a one-factor repeated measures ANOVA (baseline, 1, 2, and 4 h) was performed. Additionally, to evaluate the mephedrone effects along time of each route of administration a Dunnett multiple comparison post hoc test was conducted to compare the different time points with baseline (times 0–1 h, 0–2 h and 0–4 h) for each route of administration.

Differences in time to reach peak effects (T_max_) values were assessed using a Non-Parametric Test (Wilcoxon test).

Statistically analyses were performed using PAWS Statistics version 18 (SPSS Inc., Chicago, IL, USA). Statistically significance was defined as *p* < 0.05.

For mephedrone oral fluid concentrations, the maximum concentration (C_max_), the time needed to reach the maximum concentration (T_max_) and the AUC_0–4 h_ was calculated using the Pharmacokinetic Functions for Microsoft Excel (Joel Usansky, Atul Desai, and Diane Tang-Liu, Department of Pharmacokinetics and Drug Metabolism, Allergan, Irvine, CA, USA).

For mephedrone and metabolites urine concentrations, the amount of drug recovered in urine was calculated.

## 5. Conclusions

The study examined for the first time the acute effects of oral and intranasal mephedrone in observational naturalistic conditions. Preliminary data demonstrate that the route of administration of mephedrone could yield some appreciable differences in the acute effects attributed to mephedrone in a sample of young-adult recreational drug users. It is important to remark that each of the routes of administration carries unique and acute medical associated risks, and clinicians should be prepared to educate patients about the acute risks associated not only with mephedrone use, but also with its route of administration.

In conclusion, these results confirm that oral and intranasal mephedrone produced in natural conditions reinforcing and well-being effects in humans.

## Figures and Tables

**Table 1:** Summary of statistically significant results on the physiological and subjective effects observed after self-administration of oral (*n* = 5) and intranasal (*n* = 5) mephedrone.

Effects	Parameter	Mean ± SD		*T*-Student		ANOVA		T-Cpoints
		Oral	Intranasal	*t*	*p*-Value	*F*	*p*-Value	
Temperature	E_max_	0.4 ± 0.6	−0.2 ± 0.2	2.477	0.038			
	AUC_0–4_	0.8 ± 1.1	−0.5 ± 0.4	2.271	0.071			
	T-C					3.356	0.036	
Intensity	E_max_	48 ± 13	25 ± 17	2.376	0.045			
	AUC_0–4_	114 ± 57	37 ± 30	2.700	0.027			
	T-C					3.940	0.020	b
Stimulated	E_max_	56 ± 17	22 ± 19	2.976	0.018			
	AUC_0–4_	141 ± 56	32 ± 32	3.775	0.005			
	T-C					6.828	0.002	a, **b**
High	E_max_	65 ± 15	25 ± 17	3.952	0.004			
	AUC_0–4_	156 ± 60	33 ± 24	4.238	0.003			
	T-C					8.645	<0.001	a, **b**
Good effects	E_max_	79 ± 24	26 ± 14	4.168	0.003			
	AUC_0–4_	217 ± 101	32 ± 22	3.954	0.004			
	T-C					7.120	0.001	**a**, **b**
Liking	E_max_	83 ± 21	35 ± 15	4.110	0.003			
	AUC_0–4_	246 ± 94	62 ± 34	4.114	0.003			
	T-C					7.330	0.001	**a**, **b**, c
Content	E_max_	79 ± 25	28 ± 16	3.737	0.006			
	AUC_0–4_	238 ± 108	45 ± 37	3.766	0.005			
	T-C					8.210	0.001	**a**, **b**, c
ARCI-MBG	E_max_	12 ± 1.7	6.4 ± 2.4	4.575	0.002			
	AUC_0–4_	333 ± 14	12 ± 6.7	3.048	0.016			
	T-C					1.448	0.254	
VESSPA-SOC	E_max_	17 ± 6.0	6.6 ± 4.4	2.999	0.017			
	AUC_0–4_	46 ± 24	8.7 ± 8.7	3.237	0.012			
	T-C					8.901	<0.001	b
VESSPA-ACT	E_max_	13 ± 4.0	5.6 ± 1.5	3.679	0.006			
	AUC_0–4_	35 ± 16	7.7 ± 4.9	3.589	0.007			
	T-C					8.266	0.027	**a**, **b**, c
VESSPA-PS	E_max_	1.8 ± 1.1	0.4 ± 0.9	2.214	0.058			
	AUC_0–4_	2.6 ± 1.7	0.4 ± 0.9	2.549	0.034			
	T-C					1.567	0.223	

Emax = peak effects 0–4 h (differences from baseline); AUC_0–4_ = Area under the curve 0–4 h; T-C = temporal course 0–4 h. E_max_ measured by °C (T (temperature)) mm (visual analog scale (VAS)), and score (Addiction Research Center Inventory (ARCI), Evaluation of Subjective Effects of Substances with Abuse Potential questionnaire (VESSPA-SEE)), and expressed as mean and standard deviation. For E_max_ and AUC_0–4_, a Student’s *t*-Test for independent sample was used (see Statistical Analysis). A *p*-value < 0.05 was considered statistically significant. For T-C, a one-way analysis of variance (ANOVA) was used (see Statistical analysis). Statistical differences between oral and intranasal are presented as “a” *p* < 0.05, “**a**” *p* < 0.01 (time 1 h), “b” *p* < 0.05, “**b**” *p* < 0.01 (time 2 h), “c” *p* < 0.05, “**c**” *p* < 0.01 (time 4 h). Background color displays empty cells.

**Table 2:** Oral fluid pharmacokinetics parameters of oral (*n* = 5) and intranasal (*n* = 5) mephedrone.

Pharmacokinetic Parameters	C_max_ (ng/mL)	AUC_0–4_ (ng/mL h^−1^)	T_max_ (h)
Oral	1571 ± 1367	3684 ± 3443	2 (1–2)
Intranasal	4950 ± 5545	7917 ± 7717	1 (1–1)
*p*-value	0.296	0.373	0.083

Abbreviations: AUC: Area under the curve. SD: Standard deviation. T_max_ is shown as median (range) values.

## Data Availability

The data presented in this study are available on request from the corresponding author.

## Supplementary Materials

The following are available online at https://www.mdpi.com/1424-8247/14/2/100/s1, Figure S1: (a). Individual data of systolic blood pressure (SBP) after oral self-administration (*n* = 5); (b) Individual data of systolic blood pressure (SBP) after intranasal self-administration (*n* = 5), Figure S2: (a) Mephedrone chromatogram of a participant that self-administrated orally 150 mg of mephedrone (right) and one subject that self-administrated 50 mg intranasal (left) from and oral fluid sample (b) Chromatograms of the internal standard used for the previous samples (Mephedrone-d3), Figure S3: Linearity parameters of the oral fluid methodology, Figure S4: (a) Individual data of time-course of mephedrone oral fluid concentrations after oral self-administration of mephedrone (*n* = 5); (b) Individual data of time-course of mephedrone oral fluid concentrations after intranasal self-administration of mephedrone (*n* = 5), Table S1: Summary of time course result on the physiological and subjective effects observed after self-administration of oral (*n* = 5) and intranasal (*n* = 5) mephedrone.

## References

[1] Winstock A.R., Mitcheson L.R., Deluca P., Davey Z., Corazza O., Schifano F. (2011). Mephedrone, new kid for the chop?. Addiction.

[2] Mephedrone. https://pubchem.ncbi.nlm.nih.gov/compound/mephedrone.

[3] Papaseit E., Moltó J., Muga R., Torrens M., de la Torre R., Farré M. (2017). Clinical Pharmacology of the Synthetic Cathinone Mephedrone. Curr. Top Behav. Neurosci..

[4] Kehr J., Ichinose F., Yoshitake S., Goiny M., Sievertsson T., Nyberg F., Yoshitake T. (2011). Mephedrone, compared to MDMA (ecstasy) and amphetamine, rapidly increases both dopamine and serotonin levels in nucleus accumbens of awake rats. Br. J. Pharmacol..

[5] Simmler L., Buser T., Donzelli M., Schramm Y., Dieu L.H., Huwyler J., Chaboz S., Hoener M.C., Liechti M.E. (2013). Pharmacological characterization of designer cathinones in vitro. Br. J. Pharmacol..

[6] Troya J., Martínez de Gándara A., Ryan P., Cuevas G., Pardo V. (2019). Mephedrone and chemsex: When it stops being a party and becomes a fatal problem. Int. J. STD AIDS.

[7] Lea T., Reynolds R., De Wit J. (2011). Mephedrone use among same-sex attracted young people in Sydney, Australia. Drug Alcohol Rev..

[8] Mephedrone—An Update on Current Knowledge. https://www.drugsandalcohol.ie/12762/1/Mephedrone.pdf.

[9] Wood D.M., Dargan P.I. (2013). Novel Psychoactive Substances. Classification, Pharmacology and Toxicology.

[10] Homman L., Seglert J., Morgan M.J. (2018). An observational study on the sub-acute effects of mephedrone on mood, cognition, sleep and physical problems in regular mephedrone users. Psychopharmacology.

[11] Papaseit E., Olesti E., de la Torre R., Torrens M., Farre M. (2017). Mephedrone Concentrations in Cases of Clinical Intoxication. Curr. Pharm. Des..

[12] Busardò F.P., Kyriakou C., Napoletano S., Marinelli E., Zaami S. (2015). Mephedrone related fatalities: A review. Eur. Rev. Med. Pharmacol. Sci..

[13] Cosbey S.H., Peters K.L., Quinn A., Bentley A. (2013). Mephedrone (methylmethcathinone) in toxicology casework: A Northern Ireland perspective. J. Anal. Toxicol..

[14] Mephedrone Critical Review Report. https://www.who.int/medicines/areas/quality_safety/4_12_review.pdf.

[15] Busardò F.P., Kyriakou C., Tittarelli R., Mannocchi G., Pantano F., Santurro A., Zaami S., Baglìo G. (2015). Assessment of the stability of mephedrone in ante-mortem and post-mortem blood specimens. Forensic Sci. Int..

[16] Olesti E., Farré M., Carbó M.L., Papaseit E., Perez-Mañá C., Torrens M., Yubero-Lahoz S., Pujadas M., Pozo Ó.J., de la Torre R. (2019). Dose-Response Pharmacological Study of Mephedrone and Its Metabolites: Pharmacokinetics, Serotoninergic Effects, and Impact of CYP2D6 Genetic Variation. Clin. Pharmacol. Ther..

[17] Olesti E., Pujadas M., Papaseit E., Pérez-Mañá C., Pozo Ó.J., Farré M., de la Torre R. (2017). GC-MS Quantification Method for Mephedrone in Plasma and Urine: Application to Human Pharmacokinetics. J. Anal. Toxicol..

[18] Czerwinska J., Parkin M.C., Dargan P.I., George C., Kicman A.T., Abbate V. (2019). Stability of mephedrone and five of its phase I metabolites in human whole blood. Drug Test Anal..

[19] Busardò F.P., Gottardi M., Pacifici R., Varì M.R., Tini A., Volpe A.R., Giorgetti R., Pichini S. (2020). Nails Analysis for Drugs Used in the Context of Chemsex: A Pilot Study. J. Anal. Toxicol..

[20] Martin M., Muller J.F., Turner K., Duez M., Cirimele V. (2012). Evidence of mephedrone chronic abuse through hair analysis using GC/MS. Forensic Sci. Int..

[21] Mayer F.P., Cintulova D., Pittrich D.A., Wimmer L., Luethi D., Holy M., Jaentsch K., Tischberger S., Gmeiner G., Hoener M.C. (2019). Stereochemistry of phase-1 metabolites of mephedrone determines their effectiveness as releasers at the serotonin transporter. Neuropharmacology.

[22] Wright M.J., Vandewater S.A., Angrish D., Dickerson T.J., Taffe M.A. (2012). Mephedrone (4-methylmethcathinone) and d-methamphetamine improve visuospatial associative memory, but not spatial working memory, in rhesus macaques. Br. J. Pharmacol..

[23] Martínez-Clemente J., López-Arnau R., Carbó M., Pubill D., Camarasa J., Escubedo E. (2013). Mephedronepharmacokinetics after intravenous and oral administration in rats: Relation to pharmacodynamics. Psychopharmacology.

[24] Aarde S.M., Angrish D., Barlow D.J., Wright M.J., Vandewater S.A., Creehan K.M., Houseknecht K.L., Dickerson T.J., Taffe M.A. (2013). Mephedrone (4-methylmethcathinone) supports intravenous self-administration in Sprague-Dawley and Wistar rats. Addict Biol..

[25] Pedersen A.J., Reitzel L.A., Johansen S.S., Linnet K. (2013). In vitro metabolism studies on mephedrone and analysis of forensic cases. Drug Test Anal..

[26] Meyer M.R., Wilhelm J., Peters F.T., Maurer H.H. (2010). Beta-keto amphetamines: Studies on the metabolism of the designer drug mephedrone and toxicological detection of mephedrone, butylone, and methylone in urine using gas chromatography-mass spectrometry. Anal. Bioanal Chem..

[27] Khreit O.I., Grant M.H., Zhang T., Henderson C., Watson D.G., Sutcliffe O.B. (2013). Elucidation of the Phase I and Phase II metabolic pathways of (±)-4’-methylmethcathinone (4-MMC) and (±)-4’-(trifluoromethyl)methcathinone (4-TFMMC) in rat liver hepatocytes using LC-MS and LC-MS. J. Pharm. Biomed. Anal..

[28] Pozo Ó.J., Ibáñez M., Sancho J.V., Lahoz-Beneytez J., Farré M., Papaseit E., de la Torre R., Hernández F. (2015). Mass spectrometric evaluation of mephedrone in vivo human metabolism: Identification of phase I and phase II metabolites, including a novel succinyl conjugate. Drug Metab. Dispos..

[29] Papaseit E., Pérez-Mañá C., Mateus J.A., Pujadas M., Fonseca F., Torrens M., Olesti E., de la Torre R., Farré M. (2016). Human Pharmacology of Mephedrone in Comparison with MDMA. Neuropsychopharmacology.

[30] Papaseit E., Pérez-Mañá C., de Sousa FernandesPerna E.B., Olesti E., Mateus J., Kuypers K.P., Theunissen E.L., Fonseca F., Torrens M., Ramaekers J.G. (2020). Mephedrone and Alcohol Interactions in Humans. Front. Pharmacol..

[31] De Sousa Fernandes Perna E.B., Papaseit E., Pérez-Mañá C., Mateus J., Theunissen E.L., Kuypers K., de la Torre R., Farré M., Ramaekers J.G. (2016). Neurocognitive performance following acute mephedrone administration, with and without alcohol. J. Psychopharmacol..

[32] Czerwinska J., Jang M., Costa C., Parkin M.C., George C., Kicman A.T., Bailey M.J., Dargan P.I., Abbate V. (2020). Detection of mephedrone and its metabolites in fingerprints from a controlled human administration study by liquid chromatography-tandem mass spectrometry and paper spray-mass spectrometry. Analyst.

[33] Czerwinska J., Parkin M.C., Cilibrizzi A., George C., Kicman A.T., Dargan P.I., Abbate V. (2020). Pharmacokinetics of Mephedrone Enantiomers in Whole Blood after a Controlled Intranasal Administration to Healthy Human Volunteers. Pharmaceuticals.

[34] Jones L., Reed P., Parrott A. (2016). Mephedrone and 3,4-methylenedioxy-methamphetamine: Comparative psychobiological effects as reported by recreational polydrug users. J. Psychopharmacol..

[35] Harris D.S., Boxenbaum H., Everhart E.T., Sequeira G., Mendelson J.E., Jones R.T. (2003). The bioavailability of intranasal and smoked methamphetamine. Clin. Pharmacol. Ther..

[36] Hart C.L., Gunderson E.W., Perez A., Kirkpatrick M.G., Thurmond A., Comer S.D., Foltin R.W. (2008). Acute physiological and behavioral effects of intranasal methamphetamine in humans. Neuropsychopharmacology.

[37] Hanson L.R., Frey W.H. (2008). Intranasal delivery bypasses the blood-brain barrier to target therapeutic agents to the central nervous system and treat neurodegenerative disease. BMC Neurosci..

[38] La Maida N., Di Trana A., Giorgetti R., Tagliabracci A., Busardò F.P., Huestis M.A. (2021). A Review of Synthetic Cathinone-Related Fatalities From 2017 to 2020. Ther. Drug Monit..

[39] Papaseit E., Farré M., Pérez-Mañá C., Torrens M., Ventura M., Pujadas M., de la Torre R., González D. (2018). Acute Pharmacological Effects of 2C-B in Humans: An Observational Study. Front. Pharmacol..

[40] Papaseit E., Olesti E., Pérez-Mañá C., Torrens M., Grifell M., Ventura M., Pozo O.J., de Sousa Fernandes Perna E.B., Ramaekers J.G., de la Torre R. (2020). Acute Effects of 2C-E in Humans: An Observational Study. Front. Pharmacol..

[41] González D., Torrens M., Farré M. (2015). Acute Effects of the Novel Psychoactive Drug 2C-B on Emotions. BioMed Res. Int..

[42] Lamas X., Farré M., Llorente M., Camí J. (1994). Spanish version of the 49-item short form of the Addiction Research Center Inventory (ARCI). Drug Alcohol Depend..

[43] Martínez-Riera R., Pérez-Mañá C., Papaseit E., Fonseca F., de la Torre R., Pizarro N., Torrens M., Farré M. (2019). Soy Isoflavone Extract Does Not Increase the Intoxicating Effects of Acute Alcohol Ingestion in Human Volunteers. Front. Pharmacol..

[44] Olesti E., Pascual J.A., Ventura M., Papaseit E., Farré M., de la Torre R., Pozo O.J. (2020). LC-MS/MS method for the quantification of new psychoactive substances and evaluation of their urinary detection in humans for doping control analysis. Drug Test Anal..

[45] Olesti E., Farré M., Papaseit E., Krotonoulas A., Pujadas M., de la Torre R., Pozo Ó.J. (2017). Pharmacokinetics of Mephedrone and Its Metabolites in Human by LC-MS/MS. AAPS J..

