# Supplementary material for: Acute Pharmacological Effects of Oral and Intranasal Mephedrone: An Observational Study in Humans
Source: Pharmaceuticals (Basel). 2021 Jan 28;14(2):100. doi: 10.3390/ph14020100 (PMC7912650; doi:10.3390/ph14020100)
Supplement: Supplementary file 1 [file pharmaceuticals-14-00100-s001.pdf]

pharmaceuticals

Supplementary materials

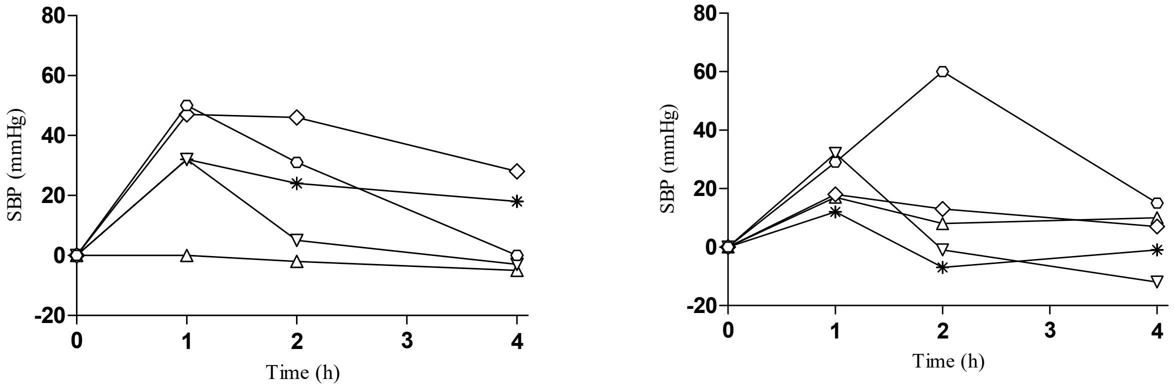

(a) (b)

Figure S1. (a). Individual data of systolic blood pressure (SBP) after oral self-administration (n=5); (b) Individual data of systolic blood pressure (SBP) after intranasal self-administration (n=5).

(a)

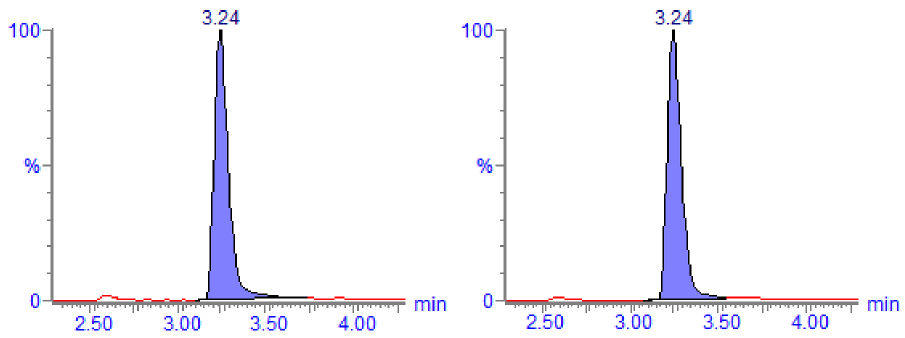

(b)

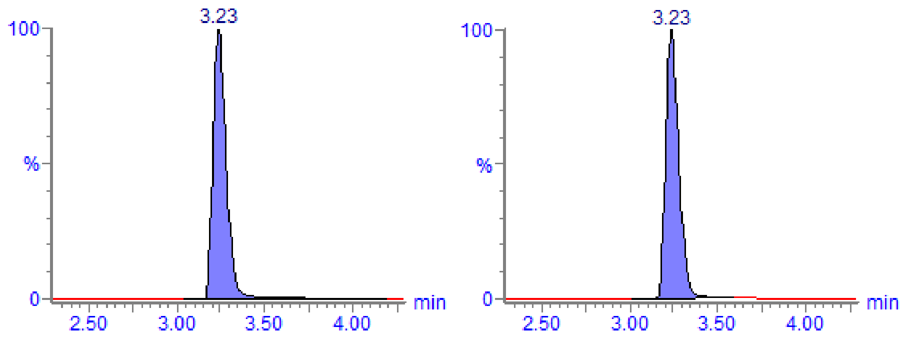

Figure S2. (a) Mephedrone chromatogram of a participant that self-administrated orally 150 mg of mephedrone (right) and one subject that self-administrated 50 mg intranasal (left) from and oral fluid sample (b) Chromatograms of the internal standard used for the previous samples (Mephedrone-d3).

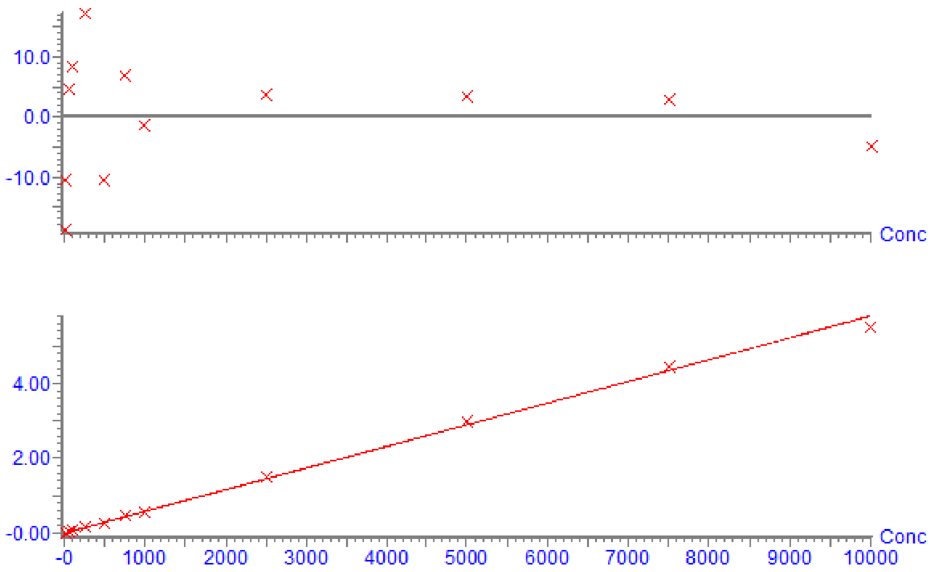

Figure S3. Linearity parameters of the oral fluid methodology.

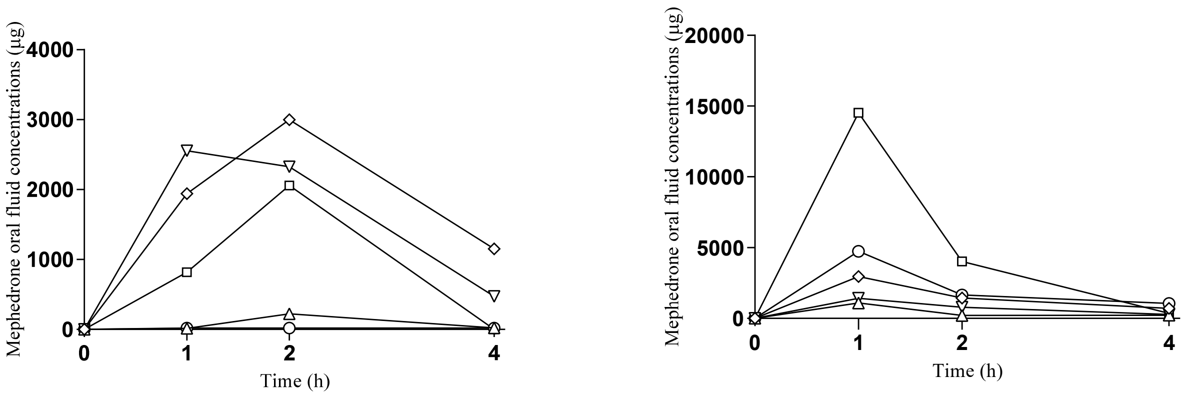

(a) (b)

Figure S4. (a) Individual data of time-course of mephedrone oral fluid concentrations after oral self-administration of mephedrone (n=5); (b) Individual data of time-course of mephedrone oral fluid concentrations after intranasal self-administration of mephedrone (n=5).

Table S1. Summary of time course result on the physiological and subjective effects observed after self-administration of oral (n=5) and intranasal (n=5) mephedrone.

	Comparison to baseline	
	Dunnett's test	
	oral	intranasal
SBP	**a**, b	NS
DBP	**a**, b	a
HR	a, **b**	**a**
T	NS	**a**
Intensity	**a**, **b**	**a**
Stimulated	**a**, **b**	**a**
High	**a**, **b**	**a**
Good effects	**a**, **b**	**a**
Liking	**a**, **b**, **c**	**a**, b
Content	**a**, **b**, **c**	**a**
Changes in colors	NS	**---**
Changes in shapes	NS	--
Changes in lights	NS	**--**
Hallucinations-seeing of lights or spots	NS	--
Hallucinations-seeing animals, things, insects, or people	NS	--
Changes in hearing	NS	NS
Hallucinations-hearings of sounds or voices	NS	--
Different body feeling	**a**	**a**
Unreal body feeling	NS	NS
Changes in distances	NS	--
Different surroundings	NS	NS
Unreal surroundings	NS	--
Confusion	NS	NS
Fear	--	NS
Depression or sadness	NS	NS
Drowsiness	NS	NS
Dizziness	NS	NS
Bad effects	NS	NS
Headache	NS	NS
Nausea	--	--
Vertigo	NS	--
Breathing difficulty	--	--
Face flushing	NS	NS
ARCI-PCAG	NS	**a**, b
ARCI-MBG	**a**, **b**	**a**
ARCI-LSD	NS	NS
ARCI-BG	**a**, b	**a**, **b**
ARCI-A	**a**, **b**, c	**a**, **b**
VESSPA-S	NS	NS
VESSPA-ANX	a, **b**	**a**, **b**
VESSPA-CP	NS	--
VESSPA-SOC	**a**, **b**	**a**

VESSPA-ACT	**a**, **b**, c	**a**
VESSPA-PS	a	NS

For T-C a post-hoc Dunnett's test for multiple comparisons was used (see Statistical analysis). Statistical differences between are presented as "a" p<0.05, "**a**" p<0.01 (times 0- 1 h), "b" p<0.05, "**b**" p<0.01 (times 0- 2 h), "c" p<0.05, "**c**" p<0.01 (times 0- 4 h).
